# Rush hour-and-a-half: Traffic is spreading out post-lockdown

**DOI:** 10.1371/journal.pone.0290534

**Published:** 2023-09-13

**Authors:** Matthew Wigginton Bhagat-Conway, Sam Zhang

**Affiliations:** 1 Department of City and Regional Planning, University of North Carolina, Chapel Hill, NC, United States of America; 2 Department of Applied Mathematics, University of Colorado, Boulder, CO, United States of America; Chang’an University, CHINA

## Abstract

Traffic congestion is ubiquitous in major cities around the world. Congestion is associated with a slew of negative effects, including delays and local air pollution. Because of the negative effects of congestion, governments invest billions of dollars into the highway system to try to reduce congestion and accommodate peak-hour automobile travel demand. The COVID-19 pandemic presented a significant disruption to transportation systems globally. One impact was a drastic reduction in travel, leading to free-flowing traffic conditions in many previously-congested cities. As lockdowns eased, traffic volumes returned to near-normal levels. However, the temporal pattern of demand may differ, due to increased remote work or other factors. In this article, we examine the temporal distribution of highway demand in California, using data from over 3,500 traffic sensors. We find that peak-hour automobile travel is spreading in the post-lockdown period. In addition to decreased traffic congestion, this finding also has implications for infrastructure investment. Roadways are generally sized based on peak-hour demand. As the peaks spread, some highway construction project may prove unnecessary. It may be possible to reallocate road space to other uses with fewer tradeoffs in terms of traffic congestion.

## Introduction

Traffic congestion is common in urban areas. Congestion is a frequent target of interventions from both traffic engineers and planners, who seek to reduce congestion for a variety of reasons. Congestion imposes significant costs on the traveling public through lost time and uncertain arrival times [1, 2]. Delayed freight shipments present a cost to businesses [2]. Congestion is associated with significant local air pollution [3], though global effects are unclear since congestion also discourages travel [4].

The COVID-19 pandemic caused massive reductions in automotive travel and thus in traffic congestion, but now traffic volumes have returned to near-pre-pandemic levels. However, congestion is caused not by overall traffic volumes, but by volumes at the peak hours. Even if traffic volumes return to pre-pandemic levels, differences in the temporal distribution of travel demand may lead to changes in congestion levels. Traffic flow is highly nonlinear. A small reduction in peak demand on a congested roadway can cause outsized reductions in traffic congestion.

In addition to congestion, reductions in peak hour auto traffic have implications for infrastructure design. Roadways are sized based on peak hour travel rather than overall travel [5]. While roadways present many economic benefits, they also have many costs. They are expensive to construct and maintain; governments in the US spent over $220 billion on highways in 2016 [6]. They present significant opportunity costs because they often occupy valuable urban land [7]. Roadway expansion may induce more driving, which presents significant negative externalities, including pollution and traffic crashes [7]. Furthermore, large roadways create barriers that disconnect communities [8]. Roadway construction also presents equity concerns. Roadway construction often displaces low-income residents and residents of color—both historically [9] and today [10]. Pollution from roadways disproportionately affects non-white communities [11].

Planners have been trying for several decades to spread out the peaks of rush-hour traffic. A number of projects in the 1970s attempted to reduce traffic congestion by staggering work start and end times [12], with varying levels of success mostly related to getting buy-in from employers. Programs in Manhattan [13] and Honolulu [14] both produced positive results.

Without an analysis of actual traffic data, it is difficult to predict from theory alone whether traffic peaks would spread out after the pandemic. On the one hand, working from home has made people’s schedules more flexible, allowing them to commute off-peak, or not at all, without majorly impacting their work life [15]. This diminishes the economic value of arriving at the office exactly at 9am. On the other hand, people are returning to in-person work [16]. Even if some people have shifted their commute schedules, historical decreases in roadway congestion have been typically associated with subsequent increases in demand that restore congestion, a phenomenon known as induced demand [17].

Studies of actual traffic data can aid in resolving these theoretical disagreements around whether peak spreading should occur, but empirical analyses of peak spreading are challenging. There is no single unambiguous mathematical definition for the idea of peak spreading. Different metrics of peak spreading can be based on different mathematical formulations, definitions of the peak, and even completely different underlying variables. Furthermore, because of the nonlinearities between speed, flow, and density, metrics that are based on different underlying variables may differ not only in magnitude but even in direction. Nonlinearities also make it difficult to differentiate peak spreading from overall changes in traffic volumes.

Roadway operators deploy networks of automated sensors across their highway networks to provide detailed, real-time information on traffic. This is a valuable data source for analysis of travel patterns, because it covers a large portion of the urban freeway network at a fine spatial and temporal resolution. However, analyses of sensor data must address issues with instability, missingness, and the computational and statistical challenges of working with large-scale and correlated data. Survey data avoid some of these issues, but introduce others—these data have smaller sample sizes, are expensive and time-consuming to collect, and may not be representative of all people or roadways.

In this article, we use data from 3,691 automated roadway sensor stations that report data at five minute intervals for over six years across the state of California, to analyze changes pre- vs. post-pandemic in the temporal distribution of roadway travel demand. To overcome the theoretical and practical issues of measuring peak-spreading on real-world sensor data, we performed our analyses on several alternative measures of peak spreading, and through a multitude of robustness checks, demonstrated that our results were not affected by missing data. We find that peak-hour travel is flattening on average, even as overall volumes near their previous levels, and we quantify important heterogeneity across sensor and location.

## Literature review

Several popular-press outlets analyzed connected-vehicle or GPS tracking data in 2021 and found that peaks were spreading. In New York and Los Angeles, July 2021 auto traffic was more temporally spread than prepandemic, with an earlier start to the evening peak and less morning travel [18]. Similarly, an analysis of four California cities found that congestion was spreading, particularly in the afternoon and evening [19]. Another analysis found rush hours shifting later and spreading nationwide [20]. A more recent study of a number of US urban areas found peak periods extending later or starting earlier in some places [21].

A stated-preference survey in Australia conducted during the lockdown found that commuters expected their arrival times at work to spread post-pandemic [22]. Spreading of peaks has also been observed on transit. On the Boston subway network, ridership has dropped more in the peaks than during the midday, though this effect was most pronounced near the start of the pandemic [23].

One likely explanation for spreading peak-hour travel demand is increasing in working from home. Telecommuting can contribute to spreading peaks in several ways. The research is unclear on whether telecommuters travel less [24] or more [25] than others, but it does seem clear that they take more non-work trips [24, 26]. These non-work trips likely occur at off-peak times.

Even on days when telecommuters do go to work, they may work from home part of the day and commute at off-peak times to avoid traffic [15, p.33]. As mentioned above, staggering work schedules is a strategy that has been deployed in the past to reduce congestion. The forecast long-term shift to a hybrid workplace may effectively lead to staggered work schedules for many workers.

## Data

**Fig 1:**
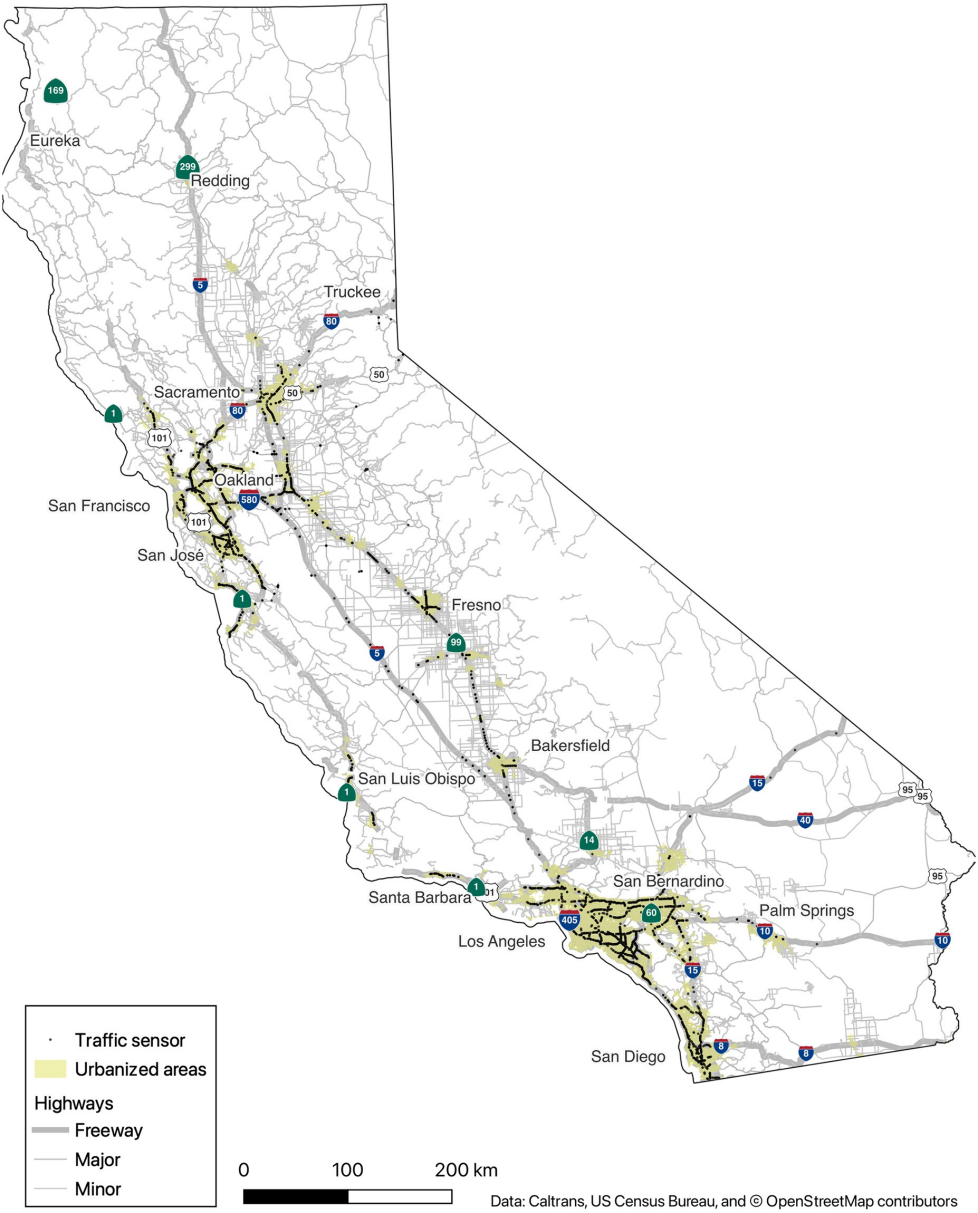
Locations of traffic sensors used in the analysis.

We obtained data from the network of roadway sensors maintained by the California Department of Transportation (Caltrans). These sensors span the state highway network, but are concentrated in urban areas and on heavily-traveled intercity routes (Fig 1). After data cleaning described below, we used data from 3,691 sensors, 82% of which were in urbanized areas, as defined by the US Census Bureau [27]. We have sensor readings at 5-minute resolution from January 1, 2016 to August 18, 2022.

Sensors exist on freeway and highway lanes as well as entry and exit ramps. Since ramps may have disparate traffic flow characteristics (for instance, due to interactions with traffic lights), we exclude them from our analysis and focus only on sensors classified as “mainline” by Caltrans. These are predominantly on freeways, but a small number occur on rural undivided highways.

Each sensor record indicated the percent of time the sensor was covered by a vehicle during that period: the *occupancy* of the sensor. The data also include *flow* (the number of vehicles that cross the sensor in a each five-minute period) and the average speed of these vehicles.

**Fig 2:**
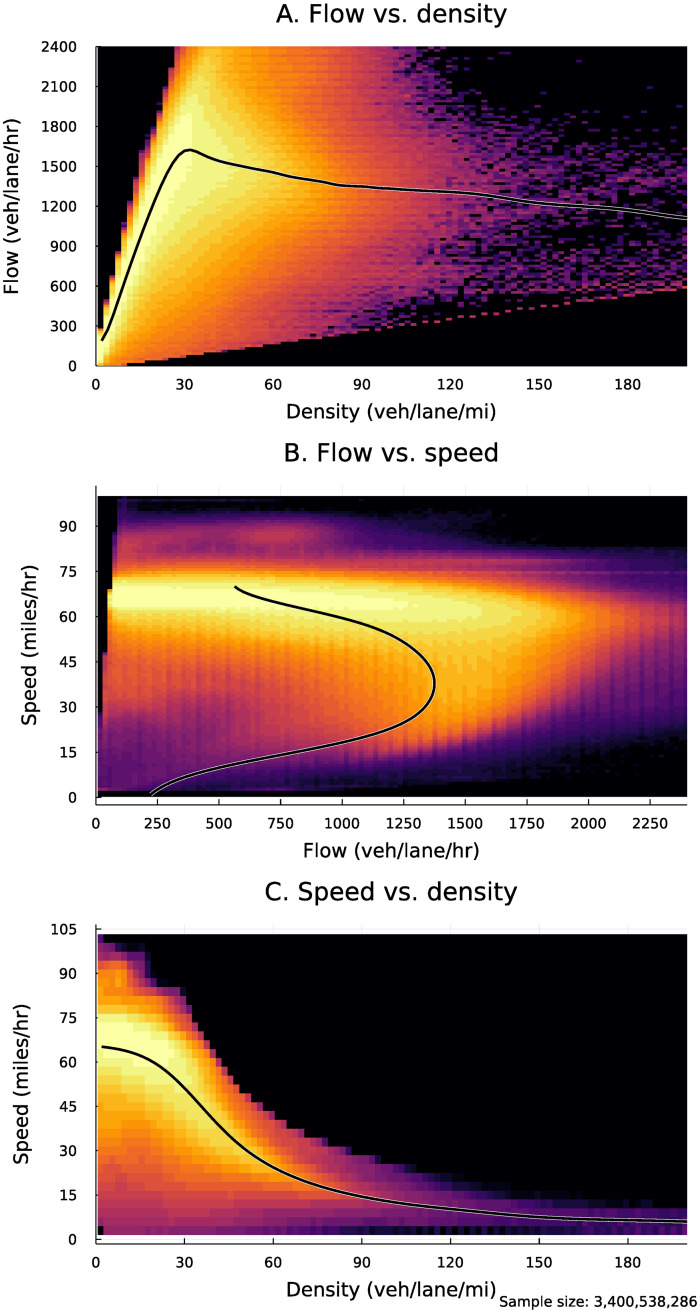
Empirical fundamental diagrams of traffic flow, showing nonlinear relationships between speed, flow, and density.

These three variables are nonlinearly and in some cases nonmonotonically related. The “fundamental diagrams” of traffic flow demonstrate these relationships. Fig 2 presents empirical versions of these fundamental diagrams, calculated from our data. The fundamental diagrams relate speed, flow, and density (vehicles per mile of roadway). While density does not appear in the sensor data, it is closely related to occupancy; the density reflects the percentage of roadway surface covered by vehicles.

Fig 2A shows the relationship between density and flow. Initially, when the road is uncongested, the density of vehicles and hourly flow of vehicles move in lockstep. However, at around 30 vehicles/lane/mile, congestion sets in. Density continues to increase, but flow increases at a decreasing rate, until eventually gridlock sets in and flow decreases as density increases.

Fig 2B shows the relationship between speed and flow. As additional motorists travel on the road, flow increases while speed remains relatively flat. Eventually congestion sets in and speeds begin to drop. Once congestion becomes sufficiently severe, flows begin to drop as well as motorists are unable to transit the roadway due to gridlock.

Finally, Fig 2C represents the relationship between density and speed. Unlike the other relationships, it is monotonic. Initially, the relationship is flat, as additional vehicles on the roadway increase density but do not affect speed. Once congestion begins to set in, speed begins to fall as density increases. Since density increases monotonically with traffic, unlike flow, we use the closely-related occupancy measure as our primary metric to assess peakiness.

Some sensors were not stable over the analysis period. We removed sensors that were not on the same highway in the same direction with the same number of lanes, or where the reported location shifted by more than 100 meters, during the analysis period or immediately thereafter. Many of these may have seen changes due to construction or reconfiguration of roadways, which could affect the results, and excluding them avoids this potential bias. We also removed sensors with no reported location in the dataset. These rules excluded 11% of sensors.

**Fig 3:**
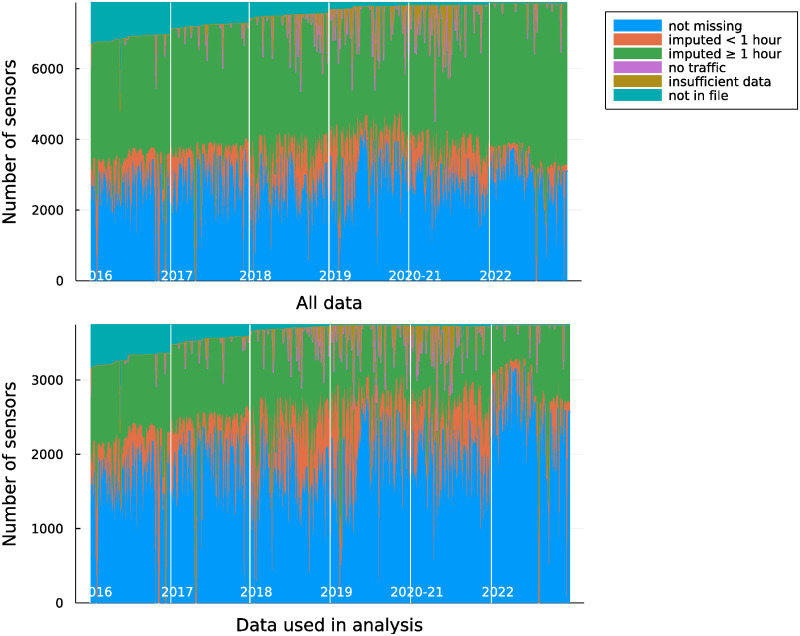
Reasons for missing data across the analysis period, for sensors that were in stable locations and road configurations over the analysis period.

There are significant missing data concerns with the sensor data. Across the prepandemic and post-lockdown periods, approximately 61% of sensor-days are imputed, at least in part, or are completely missing. Reasons for missingness over time are shown in the top panel of Fig 3. Most of the missing data has been imputed by Caltrans using a variety of reasonable methods, primarily using linear regression or median imputation [28, 29]. Sometimes only a few minutes of sensor data are missing (orange in Fig 3), but often more than an hour and in many cases the full day of data are imputed. Some detectors did not report data for all periods on a given day, and data were not imputed, which are shown in brown. A few sensors were not present in all data files from Caltrans, shown in teal. The reasons for missing data appear stationary over time, suggesting that missingness is similar in all periods and therefore less likely to bias results.

In the analysis that follows, we include nonmissing and imputed data, and drop other observations at the sensor-day level. We drop any sensors where more than 75% of observations were missing in either the pre-pandemic or post-lockdown periods. Our results are robust to and actually become stronger with changes to this threshold (Table 1). There is some variation by geography in patterns of missingness; the proportion of sensors excluded by this threshold ranges from 29% in Fresno/Bakersfield to 73% in Los Angeles.

**Table 1:** Permutation test results, overall and for selected subgroups.

	Occupancy (percentage points)		Flow (percentage points)		Entropy (centibits)		Daytime entropy (centibits)		Minutes of congestion		Number of sensors
	Δ	*p*	Δ	*p*	Δ	*p*	Δ	*p*	Δ	*p*	
Overall	-0.56	0.0	0.03	0.22	2.66	0.0	2.01	0.0	-14	0.0	3691
Urban	-0.58	0.0	0.11	0.0	2.02	0.0	1.82	0.0	-19	0.0	3013
Rural	-0.44	0.0	-0.4	0.0	5.64	0.0	2.92	0.0	13	0.0	678
**By Caltrans district**											
Sacramento (D3)	-1.18	0.0	0.07	0.01	3.2	0.0	3.2	0.0	-8	0.0	159
Stockton (D10)	0.13	0.0	-0.72	0.0	10.08	0.0	3.84	0.0	36	0.0	307
San Francisco Bay Area (D4)	-0.62	0.0	0.54	0.0	1.66	0.0	1.97	0.0	-35	0.0	919
Central Coast (D5)	0.26	0.0	0.27	0.0	-5.01	0.0	-0.34	0.0	-2	0.21	213
Fresno / Bakersfield (D6)	-0.14	0.0	-0.13	0.0	1.32	0.0	0.72	0.0	0	0.86	265
Los Angeles (D7)	-0.55	0.0	0.16	0.0	1.63	0.0	1.59	0.0	-24	0.0	508
Orange County (D12)	-1.08	0.0	-0.54	0.0	5.3	0.0	2.94	0.0	-19	0.0	648
Inland Empire (D8)	0.09	0.04	-0.16	0.0	-1.23	0.0	-0.55	0.0	37	0.0	351
San Diego (D11)	-0.97	0.0	0.27	0.0	3.92	0.0	3.39	0.0	-37	0.0	321

Some sensor-days have unreasonably-large percentages of daily occupancy happening in the peak hour, with some as large as 100%. This could be due to only a single vehicle traversing the road in a day, but is more likely due to data errors. We drop sensor-days in the top 1% of the distribution of the proportion of traffic that occurs during peak, which in this case eliminates sensor-days that report more than 22.8% of their traffic during the peak hour. We also remove any sensor-days that reported 0 vehicles over the course of the day—possibly bad data, or roads closed for construction or snow.

## Methods

**Fig 4:**
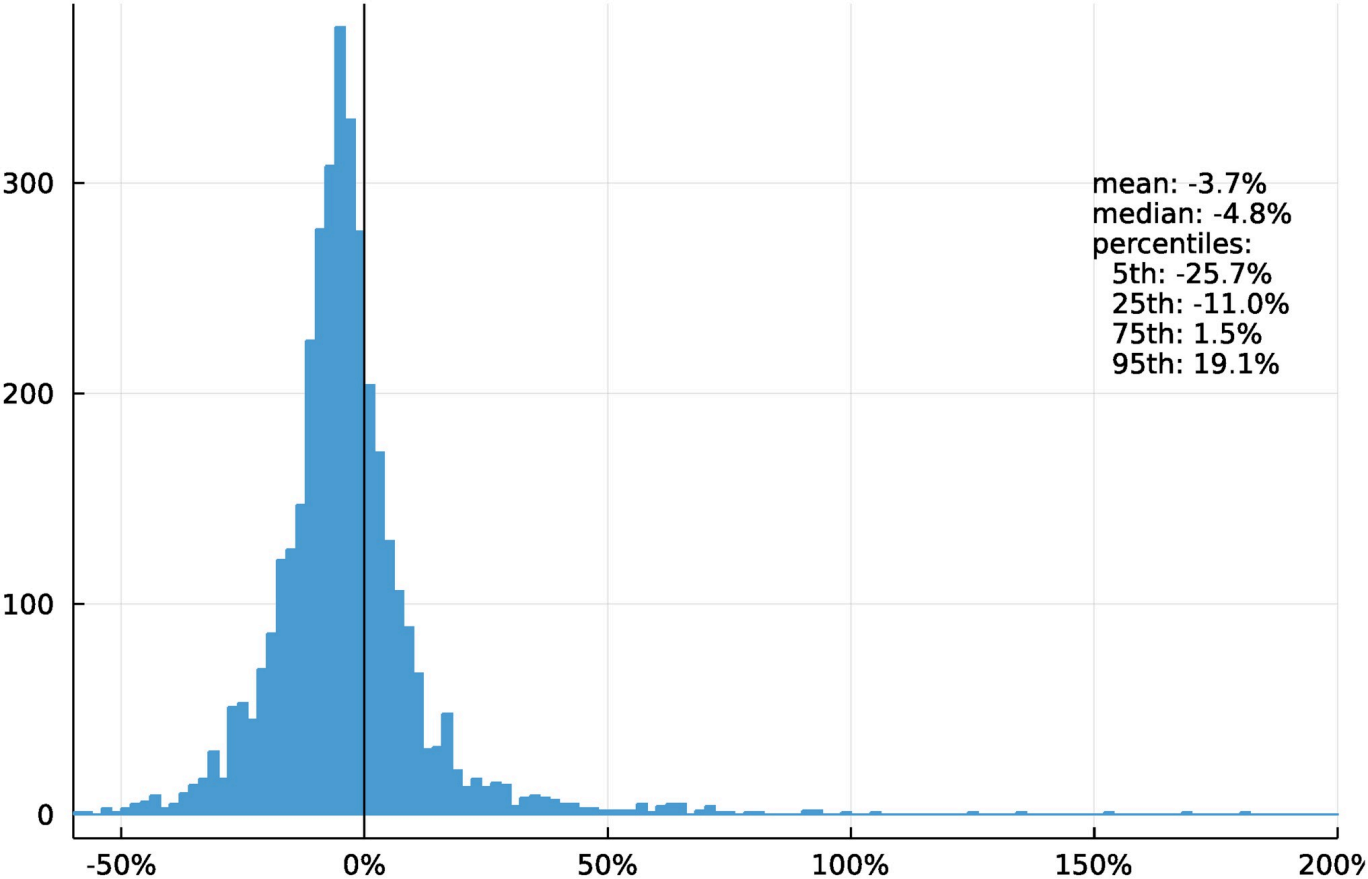
Change in mean vehicles per day, pre- to post-pandemic, by sensor.

We want to determine if traffic has become less peaked in the post-lockdown period. We define the post-lockdown period as February 16 to August 18, 2022. California’s statewide mask mandate was lifted on February 16th [30]. By this time, traffic volumes had nearly reached their pre-pandemic levels, with the mean sensor seeing 3.7% fewer vehicles per day; the median was slightly lower at 4.8% fewer vehicles (see Fig 4).

We define the pre-pandemic period as the equivalent periods in 2016–2019, starting on the third Wednesday of February and extending for 183 days. To ensure comparable seasonality effects while making sure our lockdown period was while many places were still under significant lockdowns, we split the lockdown period into two parts. We use May 3–August 20, 2020, and February 17–May 1, 2021 to avoid including prepandemic time or times when vaccines were widely available. Our results are robust to alternative specifications of the period (see S1 Appendix).

We identify the “peak hour” for each sensor-day based on the hour with the highest average occupancy. In congested areas, this does not perfectly correspond with the hour with the highest total flow, as at highly-congested times the number of vehicles passing the sensor may be constrained by congestion (in the limit, a highway that is completely stopped could have 100% occupancy across the hour but zero flow if a single car is sitting atop the sensor the entire time). Intuitively, most people think of the peak as the most congested time, which this occupancy-based definition captures.

We use four metrics of peakiness. First, we calculate the percentage of the total time the sensor is occupied over the course of the day that occurs during the peak hour. If the peaks are becoming more spread, the percentage of occupancy occurring in the peak will be reduced.

**Fig 5:**
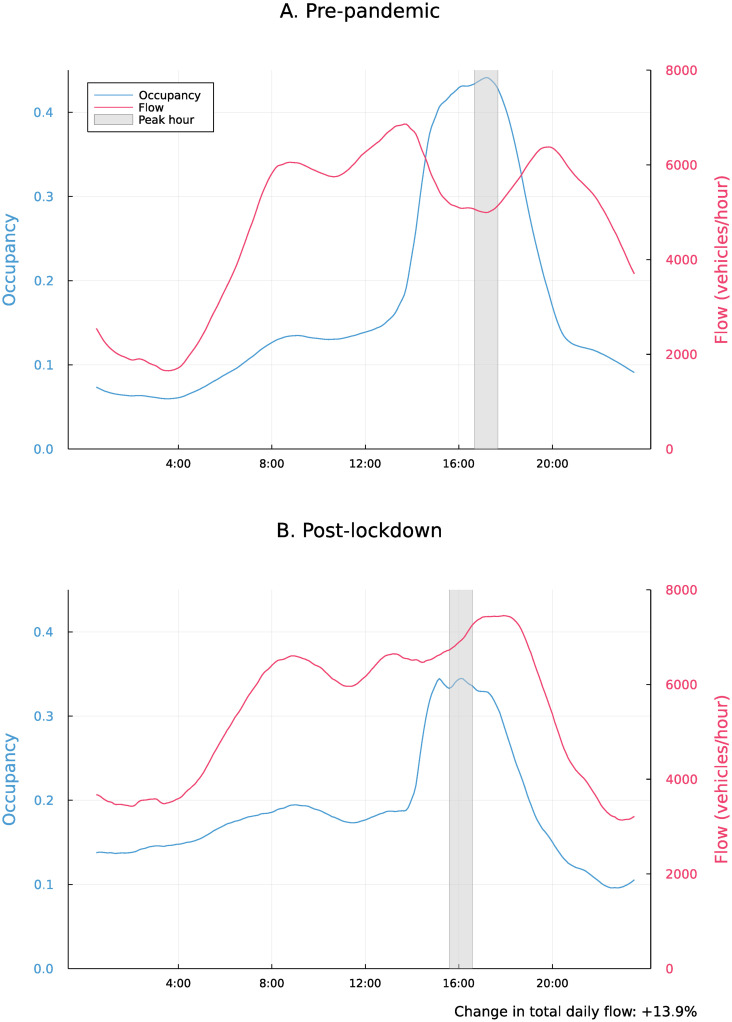
Flow and occupancy over the average day on southbound US 101 in Santa Clara, California, near the intersection with Great America Parkway.

Second, we calculate the percentage of total daily flow that occurs during the peak hour. This metric most closely matches the *k*-factor used in evaluating roadway capacity in traffic engineering [31]. This is an imperfect measure of peakiness, since the peak hour may have either higher or lower flow than other hours, depending on the level of traffic congestion. For instance, Fig 5 shows pre-pandemic and post-lockdown occupancy and flow for a freeway segment in the Bay Area over the course of an average weekday. During the pre-pandemic peak hour, the occupancy during the peak hour is high, indicating congestion. However, the flow is actually lower than other daytime hours, due to gridlock. Post-lockdown, spreading of the peaks has somewhat decongested rush hour. Even though the peaks have spread out, the reduction of gridlock has enabled a larger portion of daily vehicles to *successfully* transit the intersection during the peak hour.

Third, we calculate the entropy of the normalized occupancy over the course of the day, defined as $-\sum_{t=1}^{T}o_{t}{\text{log}}_{2}o_{t}$, where *t* is the 5-minute time period and *o*_*t*_ the proportion of daily occupancy occurring during time period *t*. This metric will be larger when traffic is more evenly spread throughout the day. Due to the nonlinearity in the logarithm, moving occupancy from a peaky time with high occupancy to a less peaked time with lower occupancy will increase the metric. We also calculate the same metric for only the daytime period (5:00 AM to 8:00 PM), since the entropy is very sensitive to changes at low-occupancy times, when we are less concerned about traffic congestion (because $\frac{d}{dx}\text{log}x$ is large when x is small).

Finally, we calculate the number of minutes of congestion, defined as traffic speeds below 50 mph (80 km/h), which is roughly the inflection point in Fig 2B. This metric is more sensitive to total daily traffic volumes than others, but provides a tangible check on our results.

We calculate each of these metrics for each sensor on the highway network for each day of the analysis period. To determine if traffic has become more or less peaky after the pandemic, we computed and compared mean values of peak-hour occupancy and entropy in the pre-pandemic and post-lockdown periods.

To determine if the observed difference in means is statistically significant, we use a permutation test [32, ch. 15]. We randomly shuffle the days assigned to the pre-pandemic and post-lockdown period 10,000 times, computing the difference in mean values for each permutation. These permutations form a sampling distribution for the difference in means under the null hypothesis of no change. We reject this null hypothesis if the observed change in means is above the 97.5^th^ percentile or below the 2.5^th^ percentile of this empirical distribution.

We randomly permute days rather than individual sensors, because of expected correlations between observations on the same day—both because many sensors observe adjacent sections of the same road, and because there may be day-level idiosyncratic effects such as weather. This block-bootstrapping methodology is commonly used for non-independent data [33].

All analysis was performed using Julia [34], Distributions [35], and Plots [36], among others.

## Results

**Fig 6:**
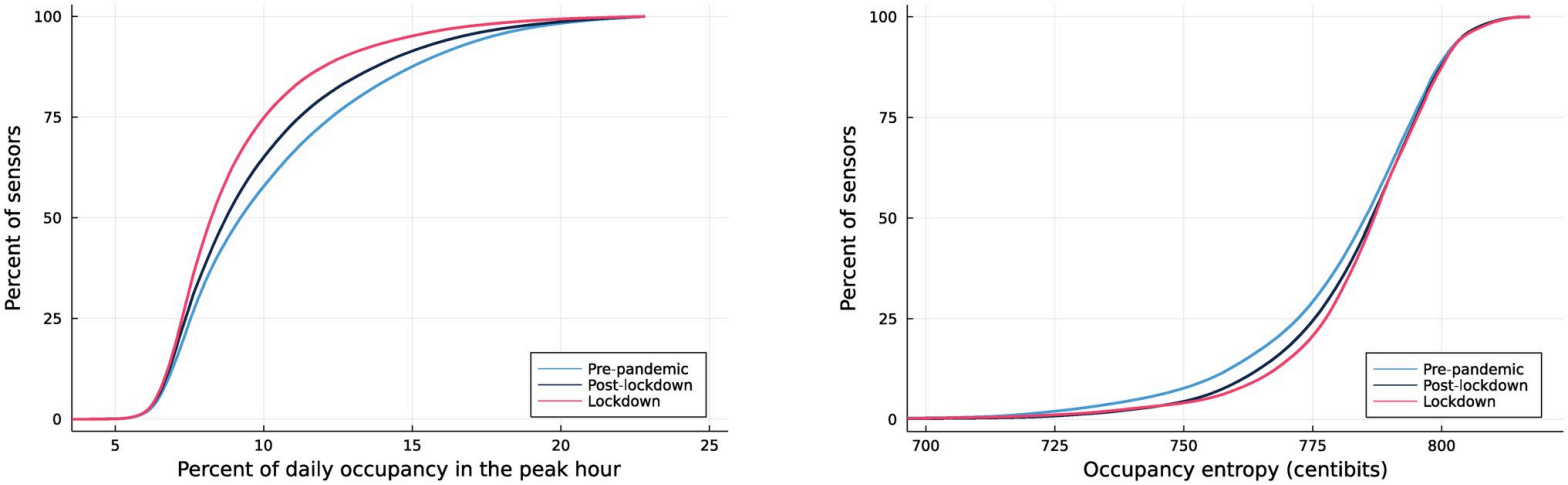
Cumulative distributions of the portion of daily occupancy occurring the peak hour (left) and entropy of occupancy (right) by period.

Our metrics suggest that the peaks indeed spread out during the pandemic, and that they have not fully returned to their pre-pandemic levels. Fig 6 shows cumulative distributions of peakiness measures before the pandemic, and during and after the lockdown. The percent of daily occupancy occurring in the peak hour shifted lower during the pandemic (left panel), and has only partially moved back towards pre-pandemic levels. The entropy shows the opposite trend, as expected, with entropy moving higher during the pandemic (indicating peak spreading), and it has only moved back a small amount (right panel).

The permutation test definitively shows that traffic is becoming more spread out, and this change is statistically significant. As Table 1 shows, the percentage of daily occupancy that occurs during the peak hour has decreased by 0.56 percentage points (a change of -5.5% from the pre-pandemic value of 10.3%), and this difference is highly significant. Entropy has increased by 0.0266 bits, and daytime entropy by 0.0201 bits, changes which are likewise statistically significant. Congestion at the average sensor has dropped by 14 minutes, a mix of peak spreading and lowered traffic volumes.

Flow, however, does not show a statistically significant difference. This is likely due to rebound effects from decreased traffic congestion in places where peak-hour gridlock previously suppressed total flow during the peak hour, as discussed above.

Daytime entropy agrees in sign with overall entropy in all tests, though magnitude varies, probably due to different traffic levels overnight when all-day entropy is very sensitive.

Where statistically significant, minutes of congestion mostly agrees in sign with the occupancy metric. The exceptions are rural areas, where the mean sensor has seen an increase in flow, and the robustness check where we specifically selected sensors that saw increased flow (see S1 Appendix). Total congestion is more sensitive to changes in flow than other metrics, explaining the discrepancy.

We performed a number of robustness checks to ensure our results are not overly sensitive to missing data, pre-existing trends, changes in overall traffic volumes, and the exact specification of the time period. None of these tests suggest different conclusions; they are presented in detail in the S1 Appendix.

### Heterogeneity

**Fig 7:**
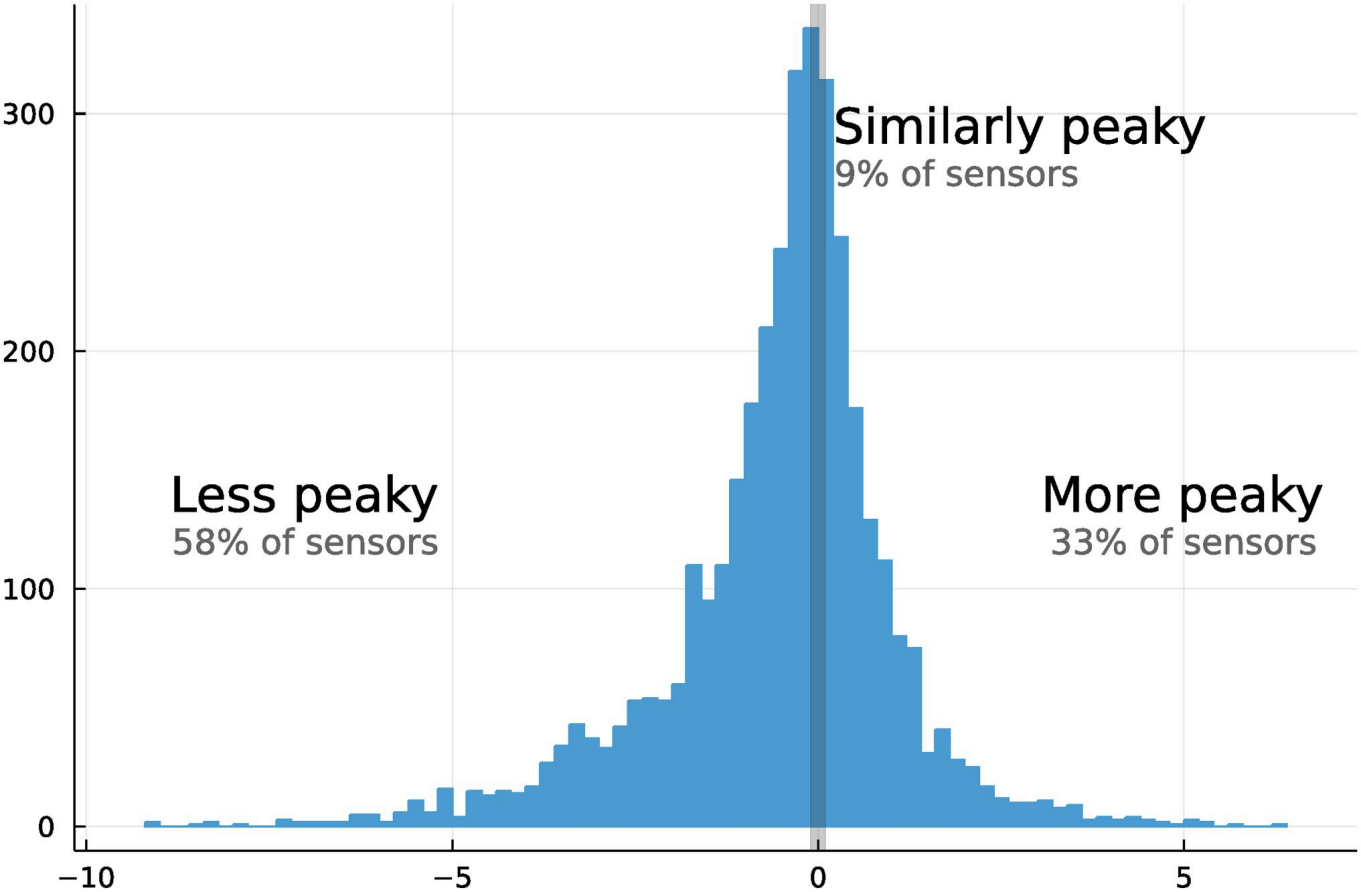
Heterogeneity in change in peakiness by sensor.

The cumulative distributions and permutation tests show clearly that peaks are spreading. However, this is not universal. Based on proportion of occupancy occurring in the peak hour, 58% of sensors see a decrease in peakiness of more than 0.1 percentage points, while 33% see an increase (Fig 7).

To understand the spatial patterns of heterogeneity in peak spreading, we computed permutation tests for urban and rural sensors separately. In rural areas, where peak-hour congestion is less common and thus peak-hour flows are less likely to be suppressed by congestion, we see the expected negative and statistically significant difference in percent of flow occurring during the peak hour. This suggests that the overall insignificant effect is related to congestion suppressing flow during peak hours in urban areas pre-pandemic.

Caltrans divides the state into a number of districts, representing the major regions of the state. In the four of the state’s largest metropolitan areas (Los Angeles, San Francisco, San Diego, and Sacramento), and in urban areas overall, peakiness of flow actually increased significantly. This is likely because flow at peak hours was significantly suppressed by traffic congestion in these metropolitan areas pre-pandemic.

Three regions show statistically significant increases in peakiness as measured by occupancy. The Central Coast is composed of smaller towns and rural areas (including Santa Barbara and San Luis Obispo), and Stockton is in the primarily agricultural San Joaquin Valley. The less urban nature of these two districts may explain their divergent trend. The Inland Empire consists of the relatively urban areas east of Los Angeles; the increase in peakiness is only marginally significant here, though it is significant as measured by entropy as well. Reasons for this are unclear and deserve further study.

## Discussion

The data clearly show that travel demand is spreading out in the post-lockdown period, even as many people’s lives start to settle into a new normal and workers return to the office. This calls into question future infrastructure investments, as many are justified based on serving peak-hour travel demand.

This may not translate to improved traffic, however. In general, increased roadway capacity leads to “induced demand” rather than reduced traffic [17]. Travelers who were driving on different routes, at different times of day, using alternative modes, or not making trips at all choose to travel on newly-enlarged roadways, often bringing congestion back to exactly where it was before. In the long term, additional roadway capacity also guides development, and further-flung areas are developed to take advantage of the increased capacity [2]. Spreading of peaks may similarly lead to induced demand—resulting a roadway system that is still just as congested at peak times.

There is one reason to believe why a capacity increase from peak spreading may not induce the same level of demand that other capacity increases do, however. For untolled roads, time spent in traffic can be considered the cost of using the road [1]. Telecommuting and flexible work arrangements are likely the main contributor to peak spreading. However, they also reduce the value of arriving at work at exactly 9 AM, and thus may reduce the amount drivers are willing to wait in traffic to do so. Since traffic only exists to the extent people are willing to wait in it, this could lead to a long-term drop in absolute congestion.

This analysis primarily relied on occupancy data, rather than the flows or vehicle counts often used in traffic engineering. Results using occupancy data show a much clearer trend than results using flow data, due to the nonmonotonic relationship between flow and congestion. Occupancy data more directly measures the experience of motorists, as high occupancy implies heavy traffic. For this reason, we encourage engineers to consider occupancy as well as flow anytime they are performing forecasting in a congested corridor.

The pandemic also resulted in a significant increase in interest for bicycling and walking [37], and many cities responded by making additional roadway space available for safe use of these modes [38]. These results suggest cities should consider making these changes permanent. As travel patterns shift, drivers may use the remaining motor vehicle lanes more efficiently, and additional lanes may not be needed to achieve acceptable performance.

Public agencies use traffic counts for a wide range of planning activities—anything from determining whether additional lanes or infrastructure such as traffic lights are needed, to whether there is space on a road to add safe bicycling facilities without causing gridlock for motorists, to what mitigations developers are required to provide for their projects. Engineers use a range of established factors to estimate peak travel demand from these counts. In the wake of the pandemic, these factors may be changing. Using pre-pandemic factors that are no longer correct could lead agencies to overbuild infrastructure, leading to increases in cost, greater climate impacts, and ultimately induce more driving due to more widely available infrastructure. Public agencies should carefully consider future expansion plans, and consider planning for lower peak demand than they might otherwise based on pre-pandemic data. Even if travel continues to increase post-pandemic, if that travel is distributed differently, additional roadway capacity may not be warranted.

The pandemic is certainly not over, and a major question left open by this research is whether this spreading of the peak will continue. We believe it will, for two reasons. First, the level of spreading has was fairly consistent in Summer 2021 and Winter 2022 (see S1 Appendix), even as people increasingly returned to their routines. If motorists were going to revert to their old behaviors post-pandemic, we would expect to see a trend in that direction, but we do not. Secondly, we believe working from home is a significant driver of peak spreading, and survey data indicates that many people expect their employers to continue to offer flexible work arrangements indefinitely [37].

## Supporting information

S1 Appendix Appendix: Robustness checks for “Rush hour-and-a-half: Traffic is spreading out post-lockdown”. (PDF)
