# Supplementary material for: Rush hour-and-a-half: Traffic is spreading out post-lockdown
Source: PLoS One. 2023 Sep 13;18(9):e0290534. doi: 10.1371/journal.pone.0290534 (PMC10499251; doi:10.1371/journal.pone.0290534)
Supplement: S1 Appendix — (PDF) [file pone.0290534.s001.pdf]

Appendix: robustness checks for "Rush hour-and-a-half: traffic is spreading out post-lockdown"

Matthew Wigginton Bhagat-Conway and Sam Zhang

August 15, 2023

We performed a number of robustness checks to ensure that our results are not caused by changes in overall traffic volumes, continuation of prepandemic trends, missing data, or the exact specification of our time period. We present results from these in Table 1 and Table 2.

It is possible that some changes in peakiness of occupancy are due to reduced overall travel demand over the course of a day. If travel demand were uniformly reduced over the course of a day on a congested corridor, occupancy metrics would show a decrease in peakiness. At uncongested times of day, occupancy would decrease roughly linearly with decreased demand, as vehicles that were occupying the sensors are removed from the roadway. However, during congested peak hours, occupancy would decrease more strongly because not only are some of the vehicles that were occupying the sensor no longer using the roadway, but those that still are are traveling faster and occupying the sensor for less time. Thus, even without any change in demand distribution, a reduction in total demand could lead to a reduction in apparent peakiness by decongesting the peaks.

To ensure that this is not what is causing the results observed here, we performed separate tests for sensors where total flow decreased and increased. If our results were driven primarily by a decrease in total flow, we would expect to see less peaky occupancy for sensors that decreased in total flow, and more peaky occupancy for those that increased. While we do find that peakiness decreased more at sensors that saw decreased flow, both subsamples show a statistically-significant reduction in peakiness. The sensors that saw a decrease in flow saw an increase in peak-hour flow, which is expected when overall traffic congestion is reduced.

We also wished to confirm that our results were not sensitive to the exact specification of the period. Some individuals and companies may not have formed a new normal immediately after the mask mandate was lifted. Thus, we also performed our analysis using a post-lockdown period of March 12–August 18; the mask mandate in K-12 schools was lifted on March 12 [1]. To ensure the result was stable over a longer period, and to account for potential seasonality effects, we also tested a "post-lockdown" period of June 15 to July 17, 2021. During this time, after vaccines became widely available and before the Delta variant surged in the US, there was no mask mandate in California [6, 5]. For each of these periods, we identified corresponding pre-pandemic periods of the same length starting on the same day of the week and week of the month in 2016–2019. Sample sizes differ slightly for each of these periods, as not all sensors were online during the different periods. Neither of these periods suggest materially different conclusions, suggesting our result is both robust and stable.

We additionally wanted to confirm that the spreading of peaks did not simply represent a continuation of a pre-pandemic trend. To test this, we created a pre-pandemic period using only days from 2019. If this were a continuation of a trend, comparing to more recent data should present a smaller change in peakiness, but the change is similar.

Table 1: Permutation test results, overall and for selected subgroups

	Occupancy (percentage points)		Flow (percentage points)		Entropy (centibits)		Daytime entropy (centibits)		Minutes of congestion		Number of sensors
	Δ	p	Δ	p	Δ	p	Δ	p	Δ	p	
Sensors that increased in flow	-0.31	0.0	-0.15	0.0	4.30	0.0	2.65	0.0	5	0.0	1076
Sensors that decreased in flow	-0.66	0.0	0.11	0.0	1.94	0.0	1.74	0.0	-22	0.0	2615
March 12-August 18 post-lockdown	-0.56	0.0	0.04	0.18	2.73	0.0	2.06	0.0	-15	0.0	3661
Summer 2021 post-lockdown	-0.6	0.0	-0.03	0.62	1.54	0.0	2.2	0.0	-10	0.0	3839
Relative to 2019 only	-0.5	0.0	0.07	0.04	2.06	0.0	1.68	0.0	-18	0.0	3691
No imputed sensor-days	-0.67	0.0	0.03	0.36	3.55	0.0	2.53	0.0	-19	0.0	3691
Sensors less than 50% imputed	-0.63	0.0	0.05	0.08	2.36	0.0	2.03	0.0	-18	0.0	2530
Sensors less than 25% imputed	-0.74	0.0	-0.01	0.75	2.74	0.0	2.22	0.0	-19	0.0	1289
Sensors less than 5% imputed	-0.75	0.0	0.06	0.1	2.23	0.0	2.09	0.0	-16	0.0	126
All sensors	-0.74	0.0	0.0	0.91	3.66	0.0	2.8	0.0	-22	0.0	7869

Lastly, we wanted evaluate whether the imputation was affecting the results. In all results discussed thus far, we drop sensors that had missing data in more than 75% of observations in either the prepandemic or postlockdown periods. We saw a similar result when removing all partially or fully-imputed sensor-days from this dataset. We also performed the test using the subsamples of sensors that had less than 50%, less than 25%, and less than 5% of their observations imputed in either period. These more stringent rules result in a stronger effect (i.e. a larger decrease in peakiness). Caltrans does not provide exact details on the time windows used to impute data; it is possible that some post-lockdown data points were imputed based on prepandemic data, thus biasing the results towards 0 when more imputed data points are included.

When including all data, imputed and observed, the results also become stronger. While the cause of this is difficult to determine exactly, our hypothesis is that it has to do with imputation methods. Caltrans uses a variety of imputation methods, with the first resort being the local regression described by Chen et al. [3]. However, for the large number of sensors that report no valid data over the entire analysis period, this method cannot be used because it requires at least some observations from the sensor being imputed, or can only be used with very old (pre-2016) data. Caltrans thus falls back to other imputation methods [2].

We additionally estimated regression models of the proportion of daily occupancy occurring in the peak hour at the sensor-day level, using the exclusion rules defined above but including all sensors up to and including those that were imputed 100% of the time. Results of these regressions are shown in Table 2. Model 1 is a simple bivariate regression. The coefficient estimate for the post-lockdown period is the difference in means between periods. This matches the estimate presented in the "all sensors" permutation test.

Model 2 adds controls for the proportion of the data for that day that was missing, in a flexible specification with 11 dummy variables. Model 3 additionally controls for traffic volumes on the day in question, and Model 4 adds sensor-level fixed effects. Including a control for the proportion of data that is missing somewhat attenuates the post-lockdown coefficient size, but the coefficient remains negative and significant, consistent with the results of the sensitivity tests above. We thus conclude that changes in missingness are not the sole driver of our results.

All models were estimated using `FixedEffectsModels.jl` [4] and use clustered standard errors at the sensor level.

Table 2: Regressions of proportion of occupancy in the peak hour on postlockdown indicator and controls

	Percent of daily occupancy in peak hour			
	(1)	(2)	(3)	(4)
(Intercept)	9.90***	10.51***	11.07***	
	(0.03)	(0.04)	(0.16)	
Post-lockdown	-0.74***	-0.54***	-0.59***	-0.63***
	(0.02)	(0.02)	(0.02)	(0.02)
Proportion imputed: 0% (exclusive)–10%		0.38***	0.37***	0.12***
		(0.04)	(0.04)	(0.01)
Proportion imputed: 10%–20%		0.31**	0.30**	0.26***
		(0.10)	(0.09)	(0.05)
Proportion imputed: 20%–30%		0.24**	0.22**	0.20**
		(0.09)	(0.08)	(0.06)
Proportion imputed: 30%–40%		0.16*	0.12*	0.25***
		(0.06)	(0.06)	(0.03)
Proportion imputed: 40%–50%		-0.08	-0.14	-0.06
		(0.08)	(0.08)	(0.05)
Proportion imputed: 50%–60%		-0.20*	-0.27**	-0.12
		(0.10)	(0.09)	(0.07)
Proportion imputed: 60%–70%		0.42**	0.31*	0.38**
		(0.14)	(0.13)	(0.13)
Proportion imputed: 70%–80%		0.25	0.01	0.61***
		(0.31)	(0.31)	(0.15)
Proportion imputed: 80%–90%		-1.38***	-1.62***	-0.32***
		(0.11)	(0.11)	(0.09)
Proportion imputed: 90%–100% (exclusive)		-1.94***	-2.06***	-0.85***
		(0.13)	(0.14)	(0.08)
Proportion imputed: 100%		-1.56***	-1.52***	-1.25***
		(0.04)	(0.04)	(0.02)
Average flow per lane (thousands of vehicles/hour)			-0.17	-0.51***
			(0.18)	(0.15)
Average flow per lane (thousands of vehicles/hour) squared			-0.10	-0.04
			(0.05)	(0.04)
Sensor fixed effects				Yes
N	4,569,230	4,569,230	4,569,230	4,569,230
R^2	0.01	0.07	0.08	0.57

References

[1] Associated Press. *California's School Mask Mandate Ends Saturday.* NBC Bay Area, 2022. URL: https://www.nbcbayarea.com/news/california/californias-school-mask-mandate-ends-thursday/2835069/ (visited on 04/28/2022).

[2] Caltrans. *System Calculations: Caltrans PeMS.* 2007. URL: https://pems.dot.ca.gov/?dnode=Help&content=help_calc#impute (visited on 07/14/2022).

[3] Chao Chen et al. "Detecting Errors and Imputing Missing Data for Single-Loop Surveillance Systems". In: *Transportation Research Record: Journal of the Transportation Research Board* 1855.1 (2003), pp. 160–167. ISSN: 0361-1981, 2169-4052. DOI: 10.3141/1855-20. URL: http://journals.sagepub.com/doi/10.3141/1855-20 (visited on 07/12/2022).

[4] Matthieu Gomez. *FixedEffectModels.Jl: Fast Estimation of Linear Models with IV and High Dimensional Categorical Variables.* Version 0.7.0. 2022. URL: https://github.com/FixedEffects/FixedEffectModels.jl (visited on 07/18/2022).

[5] Luke Money, Rong-Gong II Lin, and Melissa Hernandez. "L.A. County Will Require Masks Indoors amid Alarming Rise in Coronavirus Cases". In: *Los Angeles Times. California* (2021). URL: https://www.latimes.com/california/story/2021-07-15/l-a-county-will-require-masks-indoors-amid-covid-19-surge (visited on 10/04/2021).

[6] Deborah Netburn. "A Timeline of the CDC's Advice on Face Masks". In: *Los Angeles Times. Science* (2021). URL: https://www.latimes.com/science/story/2021-07-27/timeline-cdc-mask-guidance-during-covid-19-pandemic (visited on 10/04/2021).
